# Effects of footwear type and stair riser height on ankle inversion proprioception during stair descent in healthy young females

**DOI:** 10.3389/fbioe.2026.1911629

**Published:** 2026-10-20

**Authors:** Yangyu Chen, Jinfeng Cao, Mengde Lyu, Chengshuo Xu, Linlin Zhang, Linyan Liu, Xiang Li, Xiaoyun Tang, Jia Han, Jie Lyu

**Affiliations:** 1 School of Health Science and Engineering, University of Shanghai for Science and Technology, Shanghai, China; 2 Department of Orthopedics, Jinshan District Central Hospital Affiliated to Shanghai University of Medicine and Health Sciences, Shanghai, China; 3 College of Rehabilitation Sciences, Shanghai University of Medicine and Health Sciences, Shanghai, China; 4 Periodicals Agency of Shanghai University, Shanghai University, Shanghai, China; 5 School of Health and Biomedical Sciences, STEM College, RMIT University, Melbourne, VIC, Australia; 6 College of Medical Instruments, Shanghai University of Medicine and Health Sciences, Shanghai, China; 7 Research Institute for Sport and Exercise, University of Canberra, Canberra, ACT, Australia

**Keywords:** ankle, assessment, female, high heel shoes, proprioception, stair descent

## Abstract

**Objectives:**

To investigate ankle inversion proprioception during stair descent in healthy young females under two footwear conditions and at two riser heights.

**Methods:**

Eighteen healthy young females participated. Ankle inversion proprioception was assessed using the Ankle Inversion Discrimination Apparatus for Landing (AIDAL) during stair descent while wearing flat shoes (FS) and high heel shoes (HHS) at stair riser heights of 15 cm and 30 cm. Receiver operating characteristic (ROC) curves were plotted, and the area under the ROC curve (AUC) was calculated as the ankle proprioception score. A two-way analysis of variance (ANOVA) was employed to examine the effects of riser height and footwear type on ankle inversion proprioception.

**Results:**

A two-way ANOVA revealed a significant main effect of footwear type on ankle inversion proprioception (F = 7.513, *p* = 0.014, ηp^2^ = 0.306). No significant main effect was found for riser height (F = 0.245, *p* = 0.627, ηp^2^ = 0.014), nor was there a significant interaction effect between footwear type and riser height (F = 1.109, *p* = 0.307, ηp^2^ = 0.061).

**Conclusion:**

For the task of stair descent, FS may provide better ankle proprioceptive feedback in healthy young females compared with 7 cm HHS. It is recommended that this population consider their footwear choices in daily life based on their needs.

## 1 Introduction

Stair descent is a demanding daily activity that carries a high risk of falls and, compared with level walking, requires greater lower limb muscle control and dynamic stability (Startzell et al., 2000; Dewolf et al., 2021). In daily activities and sports, the ankle is typically the first joint to contact the ground (Brockett and Chapman, 2016). During stair descent, the human body absorbs impact energy through eccentric contractions of the knee and ankle, and therefore the ankle plays a critical role in maintaining landing stability (Brown et al., 2016; Jeon et al., 2021). Proprioception refers to the ability to perceive body segment position, movement direction, magnitude, and joint angle (Han et al., 2016). Ankle proprioception plays an important role in maintaining functional joint stability (Han et al., 2022a), and poor ankle proprioception has been identified as a critical intrinsic factor associated with an increased risk of ankle injury (Lyu et al., 2024; Herzog et al., 2019; Witchalls et al., 2012). However, ankle proprioception is not a static ability; its performance may be modulated by task characteristics and environmental factors (Han et al., 2022b; Kang et al., 2022).

In specific task contexts, riser height, as a critical environmental variable, has been demonstrated to significantly affect ankle proprioceptive ability during stair descent. For instance, Wang et al. (2025a) compared the effects of different riser heights (15 cm, 17.5 cm, and 20 cm) on ankle inversion proprioception during stair descent in individuals with and without chronic ankle instability. The results showed that as stair riser height increased, ankle proprioceptive performance significantly decreased in both populations. In addition to the geometric features of the task, the modulation of ankle function by external conditions should not be overlooked. Footwear type, as a typical human intervention factor, has primarily been studied from a biomechanical perspective; however, the resulting alterations in the joint mechanical environment may further affect sensory input and neuromuscular control processes. For example, a study by Wang et al. (2025b) showed that as the heel height of high heel shoes (HHS) increased (from 1 cm to 7 cm), the net ankle joint work, work contribution, and joint stiffness in healthy young women all decreased significantly. This may suggest that different footwear types may alter load distribution and stability control performance at the ankle. Although previous studies have demonstrated the effects of riser height on ankle proprioception under barefoot conditions, direct evidence regarding the independent role of footwear type and its interaction with riser height remains limited.

Women are the primary population who regularly wear HHS (Menz et al., 2016). Compared with men, women have a higher incidence of lower limb injuries during daily non-vehicle commuting and sports activities (Salerno and Giliberti, 2020; Talia et al., 2024). This high-risk group of women warrants attention. Furthermore, to exclude the potential influence of neuromuscular adaptations resulting from prolonged HHS wear, this study only included participants who had worn HHS for less than six hours per month over the previous two years (Gajdos et al., 2020). This study focuses on the population of healthy young females, examining ankle proprioceptive performance under shod conditions that more closely mimic daily life, serving as a complement and extension to the existing literature. Accordingly, this study aims to investigate the effects of footwear type and riser height on ankle inversion proprioception during stair descent, as well as their interaction. We hypothesized that HHS would impair ankle inversion proprioceptive performance during stair descent compared with flat shoes (FS); ankle proprioceptive performance would decline as riser height increased; and there would be an interaction effect between footwear type and riser height, such that a significant difference in proprioceptive scores between the two footwear types would be expected at a higher riser height.

## 2 Methods

This study was approved by the Committee for Ethics in Human Research at Shanghai University of Sport (approval number: 102772021RT123). All participants provided written informed consent before data collection commenced.

### 2.1 Participants

The sample size was determined using an *a priori* power analysis conducted with G*Power (version 3.1) (Faul et al., 2007), with repeated measures ANOVA selected as the statistical method. The parameters were set as follows: effect size f = 0.30, alpha level = 0.05, and statistical power (1 − β) = 0.80. The analysis indicated that a minimum of 17 participants was required, and 18 participants were ultimately recruited (age: 20.89 ± 2.63 years; height: 161.97 ± 6.78 cm; body mass: 54.35 ± 7.35 kg). Inclusion criteria were: ①female sex; ②aged 18–30 years; ③no history of lower limb surgery in the past 6 months; ④no lower limb deformities or any other conditions that could affect the ability to walk on level surfaces or stairs, and normal motor function; ⑤The monthly duration of HHS wear did not exceed six hours over the previous 2 years.

### 2.2 Instrument

According to both the Chinese and Australian national standards, the standard riser height for public residential stairs is 15 cm (Wang et al., 2025a), which also represents one of the most common heights encountered during daily walking and stair ambulation and thus has ecological validity. A riser height of 30 cm more closely approximates the heights of outdoor steps and those used in sports training (Li et al., 2026), representing an extreme riser height that may be encountered in daily life. Therefore, these two riser heights were selected in the present study, as they represent a typical environment while also enabling the observation of proprioceptive performance at different riser heights. Two types of footwear with the same forefoot sole thickness but different heel heights were selected: HHS, with a forefoot sole thickness of 1 cm and a heel height of 7 cm, and FS, in which the forefoot and heel heights were identical, both being 1 cm (Figure 1).

**FIGURE 1:**
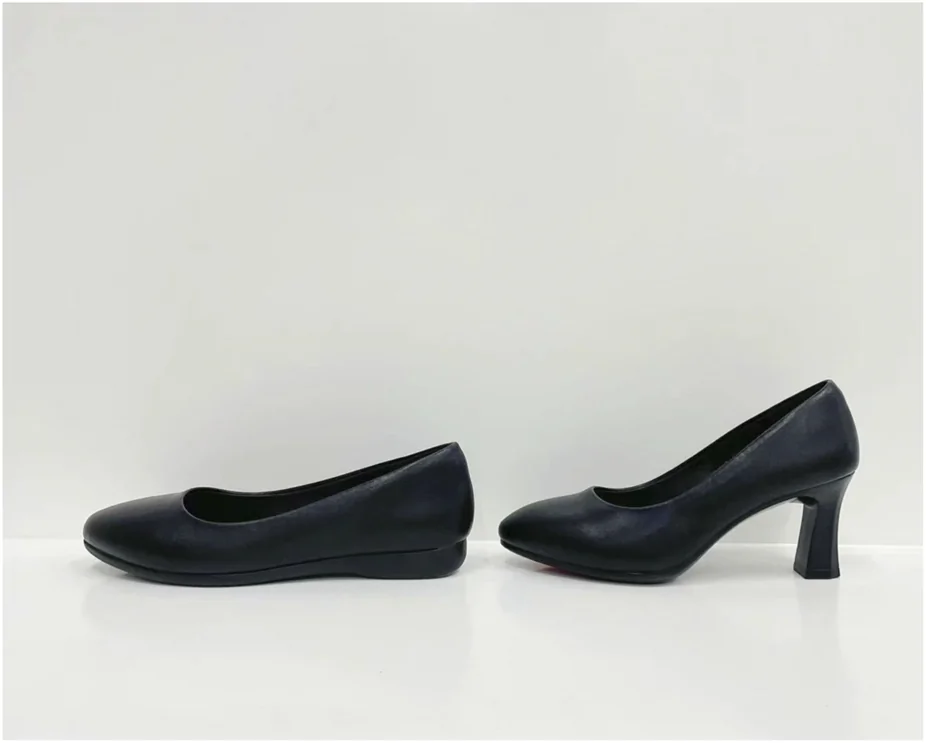
Two footwear types: flat shoes and high heel shoes.

### 2.3 Procedure

The experiment was conducted in a university biomechanics laboratory and was administered throughout by a professional examiner. To ensure sensory consistency during testing, all participants wore disposable cotton socks. Data were collected under four experimental conditions (FS and 15 cm riser height; FS and 30 cm riser height; HHS and 15 cm riser height; HHS and 30 cm riser height). All tests for each participant were completed on the same day, and the testing order was randomized by drawing cards to avoid learning effects. Owing to the apparent differences in heel height introduced by the footwear intervention, double blinding of participants and examiners was not achievable during testing. The Ankle Inversion Discrimination Apparatus for Landing (AIDAL) was used in this study (Figure 2). This apparatus was specifically developed to simulate ankle inversion angles encountered during real-world stair descent (Han et al., 2021; Wang et al., 2024). When the participants stepped onto the testing platform, it tilted to contact with the physical stop beneath it, thereby delivering preset ankle inversion angles of 10°, 12°, 14°, 16° (Shao et al., 2022).

**FIGURE 2:**
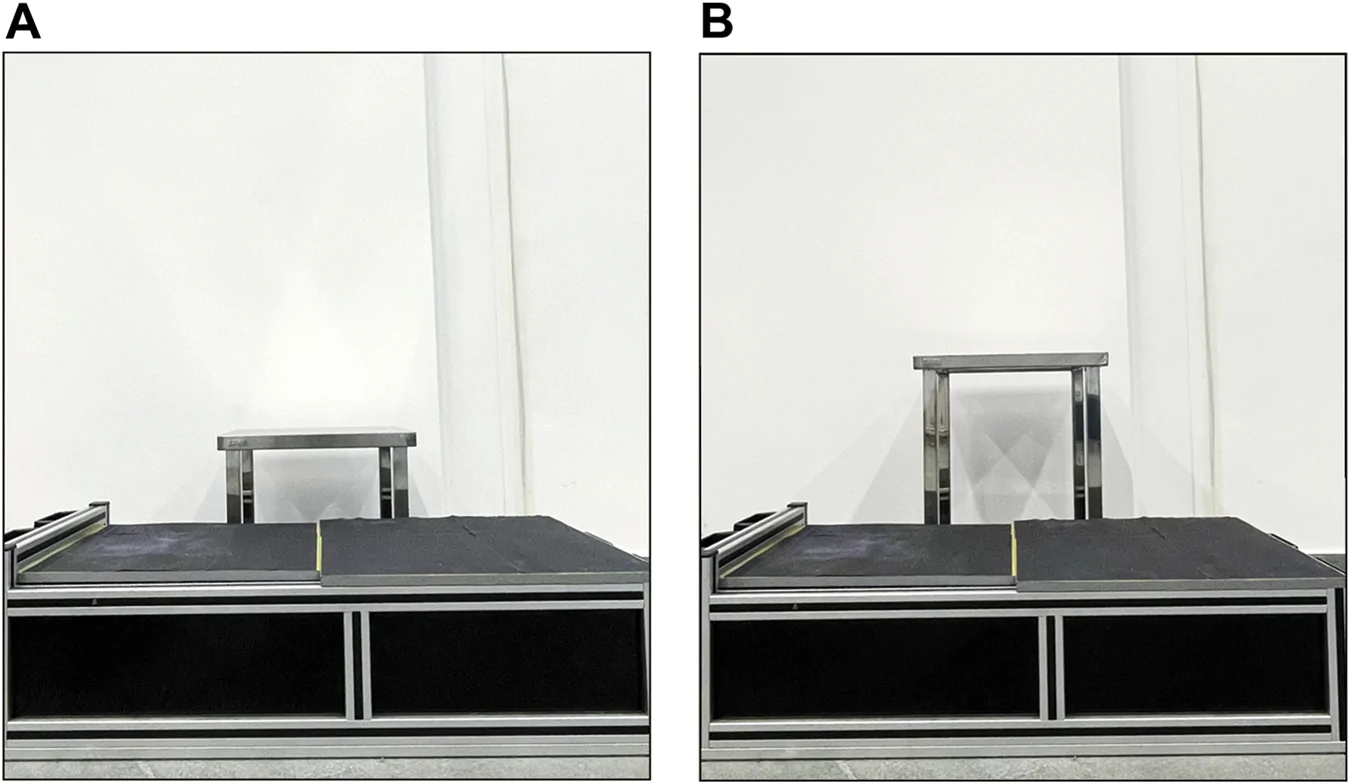
The two different heights of AIDAL. **(A)** The riser height to the proprioception testing platform is 15 cm; **(B)** The riser height to the proprioception testing platform is 30 cm.

Before proprioceptive assessment under each experimental condition, all participants first completed a familiarization session. They experienced the four different ankle inversion angles in a fixed order, with each angle experienced three times in sequence: inversion 1 = 10°, inversion 2 = 12°, inversion 3 = 14°, and inversion 4 = 16°. This process involved a total of 12 ankle inversion movements. After the familiarization, the formal testing commenced. Participants stood on the level platform wearing the test footwear, looking straight ahead with arms hanging naturally. On hearing the examiner’s “start” command, they performed the stair descent movement, stepping the dominant foot onto the wedged testing platform and the non-dominant foot onto the level ground. Once both feet were in position, they reported the number corresponding to the perceived ankle inversion angle. The dominant lower limb was defined as the leg preferred for kicking a ball (Arevalo et al., 2018). Under each combination of riser height and footwear condition, participants completed a total of 40 trials (Figure 3). To eliminate potential visual interference, they were instructed prior to testing to look straight ahead throughout the stair descent and to avoid looking down at the AIDAL. To minimize the effect of fatigue, a 10-min rest interval was provided between the four testing sessions.

**FIGURE 3:**
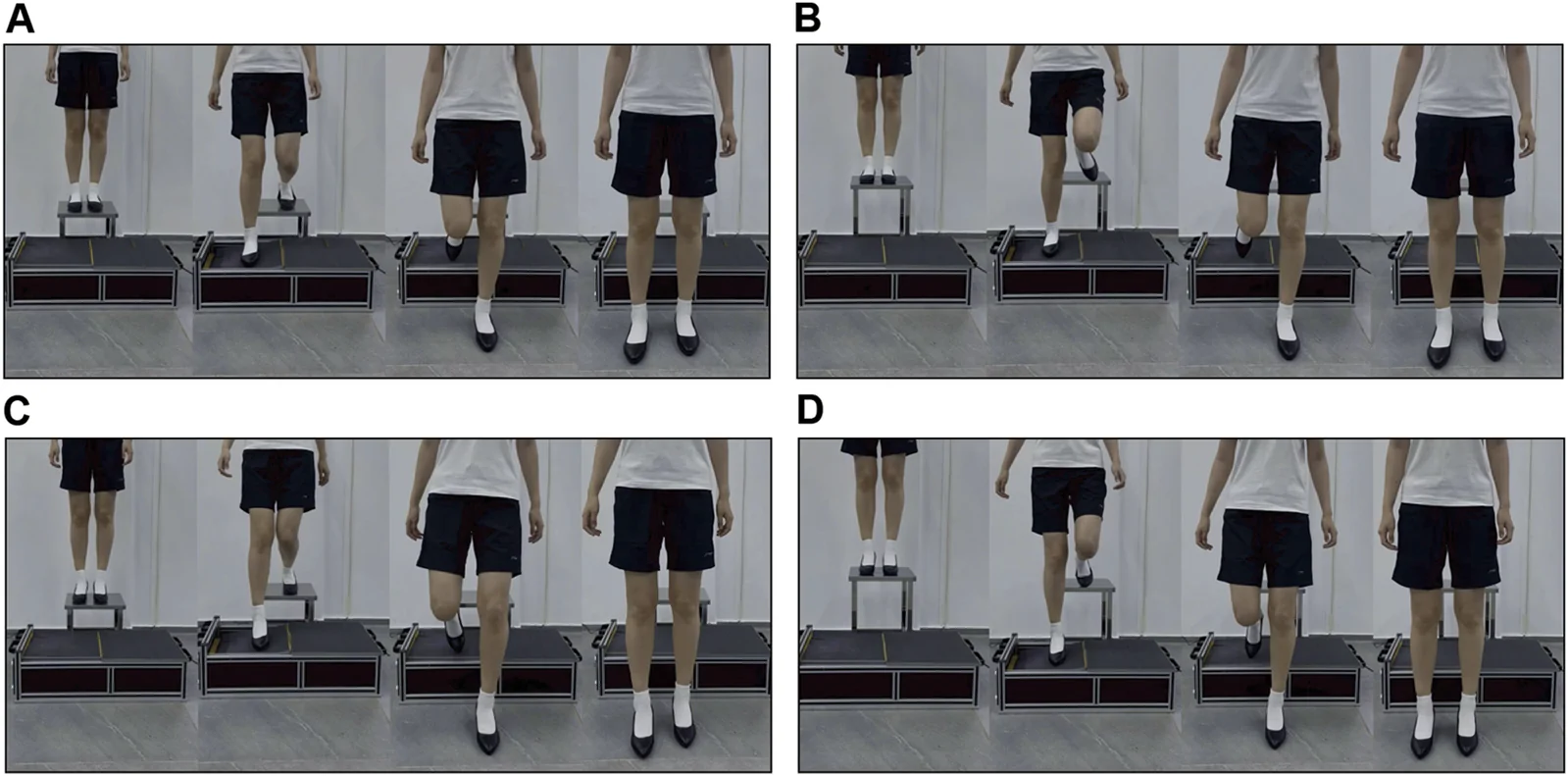
Different stair descent conditions **(A)** flat shoes and 15 cm riser height; **(B)** flat shoes and 30 cm riser height; **(C)** high heel shoes and 15 cm riser height; **(D)** high heel shoes and 30 cm riser height.

### 2.4 Statistical analysis

Descriptive data are presented as mean ± standard deviation (SD). The normality of the data distribution was assessed using the Shapiro–Wilk test. We assessed ankle proprioceptive acuity using a movement discrimination score. To derive this score, we constructed a 4 × 4 matrix recording the response frequency for each stimulus–percept pairing. Subsequently, we applied nonparametric signal detection analysis to generate three pairwise receiver operating characteristic (ROC) curves for the adjacent position pairs (1 vs. 2, 2 vs. 3, and 3 vs. 4). The mean area under the curve (AUC) of these three ROC curves was calculated, and this mean AUC value served as the final proprioceptive discrimination score (Han et al., 2021; Huang et al., 2024). AUC values can range from 0.5 (chance responding) to 1.0 (perfect discrimination). A two-way repeated measures analysis of variance (ANOVA) was conducted to compare the effects of footwear type (FS vs. HHS) and riser height (15 cm vs. 30 cm) on ankle inversion proprioception. Partial eta squared (ηp^2^) was calculated as the measure of effect size. Statistical significance was set at *p* < 0.05. All statistical analyses were performed using R statistical software (R version 4.4.3).

## 3 Results

The Shapiro–Wilk test on the model residuals confirmed that the data followed a normal distribution (W = 0.990, p = 0.823). Descriptive statistics showed that under the 15 cm riser height condition, the proprioceptive discrimination scores for FS and HHS were 0.79 ± 0.08 and 0.74 ± 0.06, respectively; under the 30 cm riser height condition, the scores were 0.77 ± 0.06 and 0.74 ± 0.08. The results of the two-way repeated measures ANOVA (Figure 4) revealed a significant main effect of footwear type on ankle inversion proprioception (F = 7.513, *p* = 0.014, ηp^2^ = 0.306). No significant main effect was found for riser height (F = 0.245, *p* = 0.627, ηp^2^ = 0.014), nor was there a significant interaction effect between riser height and footwear type (F = 1.109, *p* = 0.307, ηp^2^ = 0.061).

**FIGURE 4:**
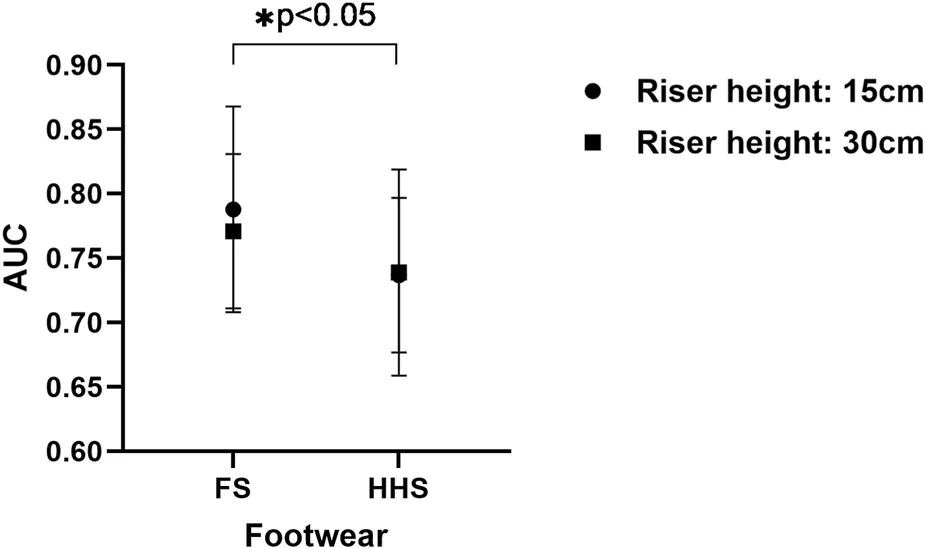
* Indicates a significant difference between footwear.

## 4 Discussion

The purpose of this study was to examine ankle inversion proprioception during stair descent in healthy young females when wearing two types of footwear at two riser heights. The results showed that ankle inversion proprioceptive performance was significantly impaired in healthy young females when descending stairs in HHS.

The results showed that ankle inversion proprioceptive performance during stair descent was significantly better when wearing FS than when wearing HHS, with a large effect size. This is consistent with our hypothesis. Previous research has shown that wearing HHS significantly alters ankle kinematic characteristics (Han et al., 2025), and such abnormal movement patterns may lead to a decline in proprioceptive performance. This outcome may be attributable to the influence of structural differences between the two footwear types on the sensory input mechanisms of the ankle. Compared with HHS, FS, by virtue of their relatively even soles, provide clearer tactile feedback from the ground and a more stable plantar pressure distribution, thereby assisting the central nervous system in accurately perceiving foot and ankle position and movement (Sun et al., 2017). In contrast, HHS elevate the heel, causing significant plantarflexion of the ankle and rearfoot, while also exacerbating foot inversion and internal rotation (Han et al., 2025). Compared with the neutral position in FS, HHS alter the initial foot position, maintaining a plantarflexed posture that keeps the calf muscles, such as the gastrocnemius, in a continuously shortened position (Beck et al., 2024). This altered state may alter the initial length–tension relationship of the muscle spindles. Meanwhile, Wang et al. (2025b) found that, compared with a 1 cm heel height, 7 cm HHS significantly reduced ankle joint net work, work contribution, and joint stiffness. The reduction in net work leads to compensatory increases in hip and knee joint work (Wang et al., 2025b). Although this strategy helps maintain overall stability, it may reduce the intensity of afferent signals from ankle proprioceptors. Reduced joint stiffness implies that the ankle joint is more susceptible to unintended inversion under weight-bearing conditions, and also lowers the baseline tension stability of mechanoreceptors such as muscle spindles (Loram et al., 2009; Hunt et al., 2019). These two factors may collectively cause the position and movement sense signals transmitted to the central nervous system to become blurred and distorted. Therefore, we speculate that the significantly poorer ankle proprioceptive performance observed with 7 cm HHS compared to FS mainly results from sensory feedback distortion caused by reduced joint stiffness and abnormal muscle spindle tension. From a proprioceptive perspective, this study further supports previous biomechanical findings. It suggests that, compared with FS, 7 cm HHS increase the risk of ankle sprain during stair descent.

We found that during stair descent in both FS and HHS conditions, proprioceptive performance did not change significantly as riser height increased. This is inconsistent with previous findings, as Wang et al. (2025a) observed a significant decline in proprioceptive acuity with increasing riser height. The discrepancy may be attributable to the inconsistency in testing conditions: Wang et al. tested barefoot, whereas we used shod conditions. The shoe sole, serving as a mechanical interface, modifies plantar sensory input, potentially attenuating afferent signals from cutaneous mechanoreceptors (Park et al., 2023). This altered sensory environment may have masked the proprioceptive demands of higher risers, thus obscuring a significant main effect. However, given that we did not directly measure the degree of sensory signal attenuation caused by the shoe sole, this interpretation lacks empirical support by direct evidence. Future research should use specific sensory assessments to empirically test the sensory attenuation effect of footwear in such motor tasks.

There was no significant interaction effect between footwear type and riser height. Previous research has shown that the neuromuscular system of healthy individuals possesses redundant capacity and that the body can better utilize this capacity as task difficulty increases (Chehrehrazi et al., 2017). During stair descent, the central nervous system of such individuals can modulate the relative contributions of the knee, hip, and ankle joints to postural control (Chiu et al., 2015). This lower-limb compensatory strategy may enable healthy individuals to maintain the proprioceptive load on the ankle at a relatively stable level when facing the challenge posed by riser heights of 15 cm and 30 cm. Of note, this compensatory strategy may be task-specific and population-specific. A recent review synthesizing lower-limb biomechanical evidence highlighted that individuals with chronic ankle instability exhibit task-specific biomechanical adaptations and neuromuscular alterations across different motor tasks (Xuan et al., 2026). This suggests that the stability observed in healthy individuals during this specific task (i.e., the absence of a significant interaction) may stem from sensorimotor compensatory redundancy; in contrast, in populations with ankle instability, this mechanism may fail due to potentially aberrant proprioceptive input, thereby manifesting as different motor control strategies. Accordingly, the consistency of footwear effects across riser heights observed in the healthy participants of the present study should be confined to this specific population and task, and should not be directly extrapolated to individuals with ankle instability. Nevertheless, this finding still indicates that, in healthy individuals, the effect of footwear type on proprioception during stair descent remains relatively stable across the two riser heights examined. However, given the relatively small sample size (*n* = 18), the statistical power may have been insufficient to detect small-to-moderate interaction effects. Therefore, the present finding that footwear effects on proprioceptive performance appeared consistent across riser heights should be considered preliminary. Future research with larger samples is needed to determine whether more subtle interactions exist between footwear type and environmental constraint.

The limitations of the present study are as follows. First, the present study did not include individuals with ankle disorders, nor did it collect biomechanical data such as ankle kinematics, plantar pressure, muscle activity, or joint stiffness, which limits the clinical generalizability of the findings. In clinical settings, the motor compensation mechanisms in individuals with ankle disorders are more complex than those in healthy individuals. For instance, recent evidence indicates that lower-limb shock attenuation and kinematic strategies deteriorate as recurrence frequency increases in patients with chronic ankle instability, and that high-frequency recurrences are associated with a maladaptive landing pattern (Wang et al., 2026). Future research should enroll populations with ankle disorders and incorporate multi-dimensional biomechanical assessments, so as to further elucidate the sensorimotor compensation mechanisms associated with ankle dysfunction. Second, ankle proprioceptive ability changes with age (Yang et al., 2019), and the participants recruited in this study were all young adults. It remains unclear whether the finding that footwear type affects ankle inversion proprioception during stair descent can be generalized to other age groups. Third, ankle proprioception in healthy populations exhibits individual differences due to varying levels of physical activity (Steinberg et al., 2019). The sample size of 18 participants may not have been sufficient to fully capture this variability. Future studies could consider expanding the sample size to evaluate the influence of this factor on ankle proprioceptive performance. Fourth, the heel height of HHS used in this study was only 7 cm. The effects of other heel heights on ankle proprioceptive acuity also warrant attention in future research. Additionally, the absence of baseline data for the barefoot control group limits the interpretation of the footwear effect. Therefore, future research should include a barefoot baseline to isolate and quantify the independent contributions of specific footwear types to proprioceptive feedback.

## 5 Conclusion

The results of this study indicate that ankle inversion proprioceptive performance during stair descent is significantly worse in healthy young females when wearing HHS than when wearing FS. For healthy young women descending stairs, FS appear to provide clearer ankle proprioceptive feedback than 7 cm HHS. Caution should be exercised when wearing HHS during such dynamic tasks, as diminished proprioceptive acuity may adversely affect ankle sensorimotor control.

## Data Availability

The raw data supporting the conclusions of this article will be made available by the authors, without undue reservation.
